# The complete mitochondrial genome of *Apis cerana*-southern China (Hymenoptera: Apidae) and insights into the phylogenetics

**DOI:** 10.3389/fgene.2025.1737945

**Published:** 2026-01-21

**Authors:** Xiang Ding, Xujiang Yu, Jing Chen, Runlang Su, Jinyou Li

**Affiliations:** 1 School of Mechanical and Electrical Information, Yiwu Industrial and Commercial College, Jinhua, China; 2 University of Chinese Academy of Sciences, Beijing, China; 3 College of Animal Science and Technology, Yunnan Agricultural University, Kunming, Yunnan, China; 4 Boston University Faculty of Computing & Data Sciences, Boston, MA, United States

**Keywords:** *Apis cerana*, Asian bee, honey bee, mitochondrial genome, phylogenetic relationship

## Abstract

The mitochondrial genome provides crucial information for understanding the evolution and phylogeny of various *Apis cerana* populations. *Apis cerana*-Southern China is a unique ecological type of Asian bee, *A. cerana*, primarily found in the coastal mountains of South China. Here, we used PacBio-HiFi sequencing to obtain the complete mitochondrial genome of *A. cerana*-Southern China and infer the phylogenetic relationships between *A. cerana*-Southern China and other *A. cerana* ecotypes. The mitochondrial genome of *A. cerana*-Southern China contains 16,137 bp and includes 13 protein-coding genes (PCGs), 22 tRNA genes, 2 rRNA genes, and an AT-rich region. The overall base composition is as follows: A (42.41%), C (9.59%), G (6.18%), and T (41.82%). The combined percentage of A and T (84.23%) is significantly higher than that of G and C. Among the 37 genes, 23 were located on the majority strand, while the remaining 14 were located on the minority strand. The phylogenetic tree based on the 13 PCGs showed that the genetic distance between *A. cerana*-Southern China, *A. cerana*-Central China, and *A. cerana*-Aba was closer. The complete mitochondrial genome sequence reported here would be useful for further phylogenetic analysis and conservation genetic studies in *A. cerana*-Southern China.

## 1 Introduction

The honey bee *Apis cerana* is an important pollinator as it provides not only pollination services but also ingredients for healthy human foods (Chen et al., 2017). However, the populations of *A. cerana* have declined significantly in recent decades due to climate change, human activities, and bee diseases (Cao et al., 2020; Li et al., 2022; Matias et al., 2017). Mapping its genetic variation is crucial for understanding population-level health, histories, and the potential capacity to respond to environmental changes. Thus, the study of genetic diversity is fundamental to the conservation and utilization of honey bee resources, and it is particularly crucial for *A. cerana* populations that are experiencing rapid declines. Therefore, it is important to elucidate the mechanisms of *A. cerana* environmental adaptation with sensitive morphological characteristics or molecular markers.

Assessments of honey bee genetic diversity have included morphological comparisons and the identification of molecular markers. Relying solely on morphological characteristics to differentiate *A. cerana* groups comes with limitations, but the use of molecular markers is currently recognized as a more reliable and accurate method (Li et al., 2019). Molecular genetic markers are one of the most powerful tools for genomic analysis, linking inherited traits to potential gene variants by locating and identifying heritable DNA sequences (Duran et al., 2009). The main molecular genetic markers commonly used in honey bees include restriction fragment length polymorphisms (RFLP), randomly amplified polymorphic DNA (RAPD), repeated simple sequences (SSR), single nucleotide polymorphisms (SNP), and mitochondrial DNA (mtDNA) markers (Qamer et al., 2021; Syromyatnikov et al., 2018; Wang et al., 2022).

At present, mtDNA markers are among the most frequently used molecular marker techniques. Due to their rapid rate of evolution, simple structural composition, and abundant genetic information, mitochondria have become crucial for studying the evolutionary origins and genetic diversity of organisms (Huang et al., 2021; Mandal et al., 2014; Wang et al., 2020). *A. cerana* exhibits a consistent matrilineal mitochondrial pattern, it is considered as an ideal organism for studying genetic diversity through the mitochondrial genome. The mitochondrial genome provides crucial information for mastering molecular evolution and phylogeny. The evolution of the *A. mellifera* mitochondrial genome has revealed genomic differences in *A. mellifera* at the subspecies level (Eimanifar et al., 2018). An evolutionary analysis of *A. cerana* from China and Nepal using *COI-COII* sequences showed that genetic differentiation was co-induced (Tan et al., 2016). Previous research has shown that among honey bees, the mitochondrial genome differs by species, subspecies, and geographical groups. The mitochondrial genome can be used to explore the genetic diversity and evolution of *A. cerana* populations (Tang et al., 2023).

In this study, we report the complete mitochondrial genome of *A. cerana*-Southern China. The analysis of the sequence and structure provides valuable insights into the possible mutations in the mitochondrial genome of honey bees to a large extent. It also sheds light on the evolution and environmental adaptation of *A. cerana*. This study can provide important references for the conservation and utilization of honey bee germplasm resources, as well as for the breeding and evolution of honey bees.

## 2 Materials and methods

### 2.1 Sample collection and DNA sequencing

The *A. cerana*-Southern China samples were obtained from the Hongchang Town, Chaonan District, Shantou, Guangdong Province, China (N 23.31, E 115.95). Fifty drone pupae were collected from one colony, then placed in absolute ethanol and stored in a −20 °C freezer. The genomic DNA was extracted and sent to Berry Genomics Co., Ltd. (Beijing, China) for PacBio HiFi sequencing.

### 2.2 Mitochondrial genome assembly and annotation

PacBio HiFi reads generated for *A. cerana*–Southern China were first used to assemble the nuclear genome, including mitochondrial sequences, using Hifiasm v0.19.5-r587 (Cheng et al., 2021; Cheng et al., 2022). The resulting contigs from the Hifiasm assembly were indexed to construct a local BLAST database. A reference *A. cerana* mitochondrial genome (GenBank accession NC_014295) was downloaded and used solely to identify mitochondrial contigs within the Hifiasm assembly by BLAST searches, rather than for genome assembly. The identified mitochondrial contigs were then extracted for downstream analyses. Preliminary annotation of the mitochondrial genome was performed using the MITOS Web Server (Bernt et al., 2013). The annotation results were subsequently refined and validated using GeSeq implemented in Chlorobox (Tillich et al., 2017) by comparison with previously reported protein-coding and RNA genes from related species. A circular map of the mitochondrial genome was generated using Chloroplot (Zheng et al., 2020). The secondary structures of tRNA genes were predicted using the MITOS Web Server under the invertebrate mitochondrial genetic code (Bernt et al., 2013), and the tRNA secondary structure diagrams were manually redrawn using Adobe Illustrator.

### 2.3 Mitochondrial genome sequence analysis

BioEdit (version 7.0.9.0) (Hall, 1999) was used to calculate the base composition and codon usage of the complete *A. cerana*-Southern China mitochondrial genome sequence and the protein-coding genes (PCGs), tRNA genes, rRNA genes and AT-rich regions. Codon usage bias, represented by relative synonymous codon usage (RSCU), was calculated using codonW software (retrieved from https://codonw.sourceforge.net/) and was plotted into a bar graph using JSHYCloud (http://cloud.genepioneer.com:9929). The RSCU greater than 1 indicated a preference for certain amino acids. The AT and GC biases were calculated according to the following formulas: AT skew = (A-T)/(A + T) and GC skew = (G-C)/(G + C).

### 2.4 Phylogenetic analyses

**FIGURE 1:**
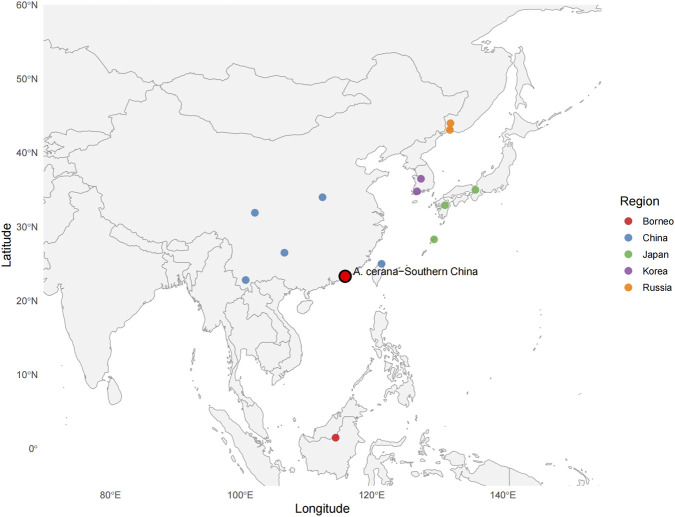
Geographic distribution of *Apis cerana* samples used for phylogenetic analysis.

The mitochondrial genome of *A. cerana*–Southern China, assembled from PacBio HiFi sequencing data, has been deposited in the NCBI GenBank database under the accession number PP692293. In addition to this newly generated mitogenome, a total of 18 honey bee mitochondrial genomes were included in this study, comprising 15 previously published sequences retrieved from GenBank. The mitochondrial genomes of *Apis mellifera* sinisxinyuan and *Apis mellifera* capensis were selected as outgroups (Table 1). Because mitochondrial tRNA and rRNA genes are highly conserved in both nucleotide sequences and secondary structures, their inclusion provides limited phylogenetic resolution. Therefore, 13 mitochondrial protein-coding genes (PCGs) were used to infer the phylogenetic relationships among *A. cerana* populations from different geographic regions (Figure 1).

**TABLE 1:** Information about the sequences of phylogenetic tree.

Number	Species	GenBank accession number	Country
1	*Apis cerana japonica*-Amami	*AP017941.1*	Japan
2	*Apis cerana koreana*-South Korea	*MW309837.1*	Korea
3	*Apis cerana japonica*-Kyoto	*AP017314.1*	Japan
4	*Apis cerana japonica*-Tsushima	*AP017985.1*	Japan
5	*Apis cerana*-Korea	*KX908206.1*	Korea
6	*Apis cerana*-Primorsky Territory	*LC640350.1*	Russia
7	*Apis cerana*-Vladivostok	*AP018450.1*	Russia
8	*Apis cerana koreana*-Jeollanamdo	*AP018431.1*	Korea
9	*Apis cerana*-Central China	*AP017983.2*	China
10	*Apis cerana*-Aba	*OP689704.1*	China
11	*Apis cerana*-South Yunnan	*NC_014295.1*	China
12	*Apis cerana*-Taipei	*AP017984.2*	China
13	*Apis cerana*-Borneo	*AP018149.1*	Borneo
14	*Apis cerana*-South Yunnan	*PP175371*	China
15	*Apis cerana*-Yungui plateau	*PP175370*	China
16	Apis mellifera sinisxinyuan*	*MN733955.1*	China
17	Apis mellifera capensis*	*MG552693.1*	South Africa

The asterisk represents the outgroup.

The 13 PCG sequences were input into PhyloSuite (V1.2.3) software (Xiang et al., 2023; Zhang et al., 2020) and aligned using MAFFT. Then, the PCG sequences were concatenated. Modelfind was used for evolutionary model selection with a BIC value. The evolutionary tree was constructed using MrBayes and an IQ tree with default parameters.

### 2.5 Chromosome anchoring and mito–nuclear synteny analysis

The PacBio HiFi–assembled nuclear genome was anchored to chromosomes using RagTag (Alonge et al., 2022) against the *Apis cerana* chromosome-level reference (*GCA_029169275.1*), after which the complete mitochondrial genome served as the BLASTn query to assess mito-nuclear collinearity (synteny).

## 3 Results

### 3.1 Mitogenome organization and base composition

Using PacBio HiFi sequencing data, we assembled a high-quality mitochondrial genome of *A. cerana*–Southern China (GenBank accession PP692293). PacBio HiFi sequencing generated 64.44 Gb of high-quality long-read data from drone pupae, with an average read length of approximately 17.8 kb and a mapping ratio of 99.90%, providing sufficient coverage for reliable genome assembly. The Hifiasm assembly produced a high-quality nuclear genome (total length: 231.9 Mb) with 40 contigs and a contig N50 of 11.78 Mb, supported by a BUSCO completeness score of 97.90%.

**FIGURE 2:**
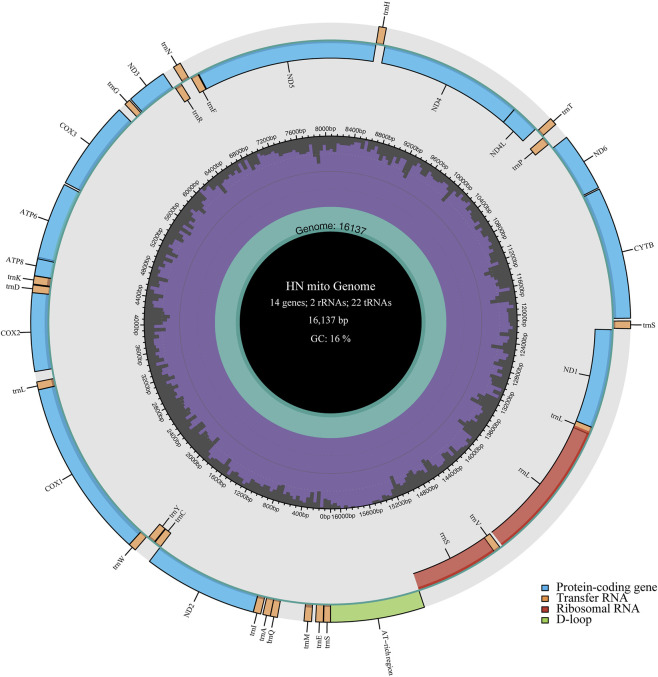
Graphical maps of the mitochondrial genome of *A. cerana*-Southern China. The inside circles show the G + C content.

We assembled the complete *A. cerana*–Southern China (GenBank: PP692293) mitochondrial genome (Figure 2). The complete mitochondrial genome is 16,137 bp in length and contains 37 genes, including 13 protein-coding genes (PCGs), 22 tRNA genes, two rRNA genes, and an AT-rich region (Table 2). Among these genes, 23 are located on the J strand (majority strand), whereas the remaining 14 are encoded on the N strand (minority strand) (Figure 2; Table 2). The nucleotide composition of the complete mitochondrial genome is A = 42.41%, T = 41.82%, C = 9.59%, and G = 6.18%, resulting in a high A + T content of 84.23%. Most protein-coding genes (PCGs) initiated with standard ATN start codons, while several genes used TTA as an alternative initiation codon. In several genes, including ND5, ND4, ND4L, and ND1, TTA is annotated as an alternative initiation codon, based on conserved open reading frames and comparative alignment with closely related Apis mitochondrial genomes.

**TABLE 2:** Annotations of the mitochondrial genome of *A. cerana*-Southern China.

Name of gene	Start position	End position	Length/bp	Intergenic nucleotides/bp	Initiation codons	Termination codons	Direction
trnS1(Ser)	1	60	60	​	​	​	J
trnE (Glu)	64	129	66	3	​	​	J
trnM(Met)	164	229	66	34	​	​	J
trnQ (Gln)	459	520	62	229	​	​	J
trnA (Ala)	521	586	66	0	​	​	J
trnI(Ile)	605	670	66	18	​	​	J
*ND2*	671	1666	996	0	ATT	TAA	J
trnC(Cys)	1666	1731	66	−1	​	​	N
trnY (Tyr)	1737	1805	69	5	​	​	N
trnW (Trp)	1822	1890	69	16	​	​	J
*COX1*	1891	3456	1566	0	ATT	TAA	J
trnL2 (Leu)	3452	3521	70	−5	​	​	J
*COX2*	3611	4291	681	89	ATT	TAA	J
trnD (Asp)	4290	4357	68	−2	​	​	J
trnK (Lys)	4364	4435	72	6	​	​	J
*ATP8*	4442	4603	162	6	ATC	TAA	J
*ATP6*	4585	5262	678	−19	ATG	TAA	J
*COX3*	5280	6059	780	17	ATG	TAA	J
trnG (Gly)	6128	6193	66	68	​	​	J
*ND3*	6194	6547	354	0	ATT	TAA	J
trnR (Arg)	6567	6632	66	19	​	​	N
trnN(Asn)	6652	6719	68	19	​	​	J
trnF(Phe)	6738	6808	71	18	​	​	N
*ND5*	6815	8482	1668	6	TTA	TAA	N
trnH(His)	8484	8549	66	1	​	​	N
*ND4*	8567	9895	1329	17	TTA	TAT	N
*ND4L*	9896	10159	264	0	TTA	AAT	N
trnT (Thr)	10183	10249	67	23	​	​	J
trnP(Pro)	10265	10343	79	15	​	​	N
*ND6*	10393	10905	513	49	ATT	TAA	J
*CYTB*	10918	12066	1149	12	ATG	TAA	J
trnS2(Ser)	12090	12156	67	23	​	​	J
*ND1*	12169	13083	915	12	TTA	AAT	N
trnL1 (Leu)	13084	13152	69	0	​	​	N
Large subunit rRNA (*lrRNA*)	13131	14454	1324	−22	​	​	N
trnV(Val)	14480	14546	67	25	​	​	N
Small subunit rRNA (*srRNA*)	14547	15319	773	0	​	​	N
AT-rich region	15320	16137	818	0	​	​	J

Regarding termination codons, most PCGs terminate with the conventional mitochondrial stop codon TAA. In a small number of cases, truncated stop codons were inferred, which are presumed to be completed through post-transcriptional polyadenylation, a common mechanism in insect mitochondrial genomes. The intergenic regions of the *A. cerana*–Southern China mitochondrial genome range from 0 to 229 bp in length.

### 3.2 Protein-coding genes

**FIGURE 3:**
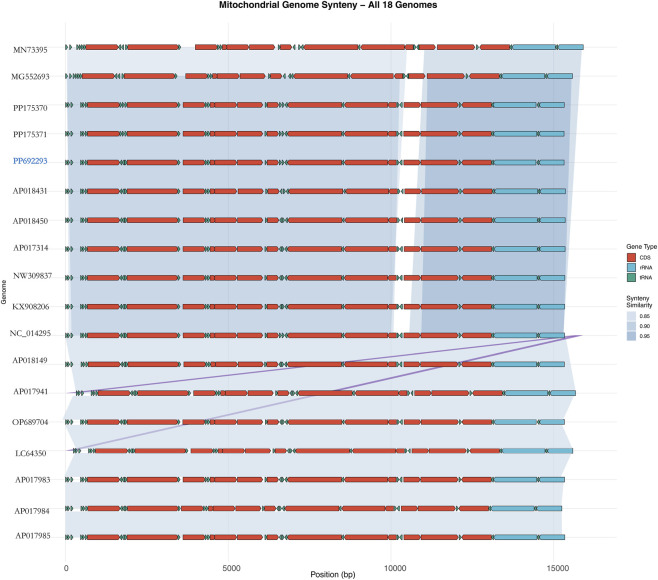
Synteny of 13 mitochondrial protein-coding genes across 18 genomes.

**FIGURE 4:**
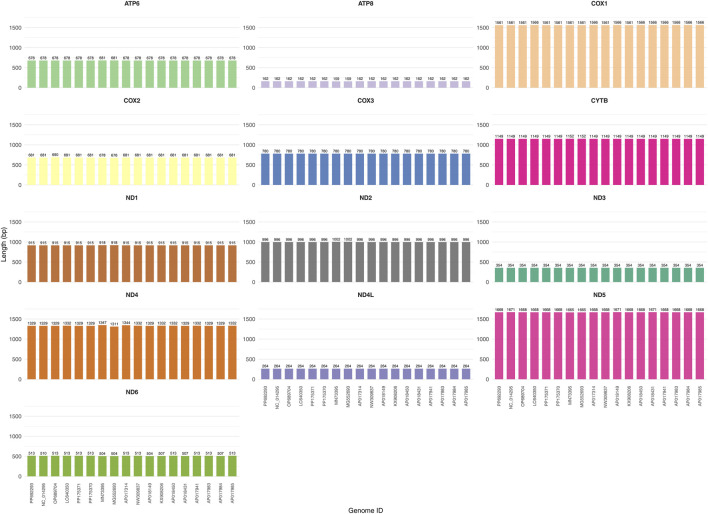
Length variation of the 13 mitochondrial protein-coding genes across 18 mitogenome.

The total length of the 13 PCGs in *A. cerana*-Southern China was 11,055 bp, accounting for 68.51% of the entire mitochondrial genome (Table 3). The length and base composition of the PCGs were varied, the 13 PCGs ranged from 162 bp (*ATP8*) to 1668 bp (*ND5*). Except for the A + T content of *COX1* and *COX2*, the A + T content of the other PCGs was greater than 80%, indicating an AT preference. According to the AT-skew and GC-skew results, most of the PCGs were biased toward T, while all PCGs showed a preference for codon C. We conducted an analysis of the frequency and RSCU values for 13 PCGs in the *A. cerana*-Southern China mitochondrial genome. The RSCU values for the 13 PCGs were greater than 1, except for those of the two amino acids Met and Trp, which were each encoded by only one codon (Figure 2). This suggests that all codons were biased. The TTA codon had the highest RSCU value in *A. cerana*-Southern China (3.84), followed by the AGA codon (3.46). NNA-type codons accounted for 41.93% of the total number of codons, indicating that codons with an A at the third position were the most frequent. This study conducted a synteny analysis of the 13 protein-coding genes (PCGs) across 18 mitochondrial genomes (Figure 3). The results show that the order and strand orientation of the PCGs are highly conserved among genomes, with overall synteny similarity frequently reaching ≥0.95 and no large-scale rearrangements that would alter the ordering of coding regions. The differences observed are largely confined to noncoding hypervariable regions or a few tRNA clusters, manifested as changes in intergenic spacer length and minor shifts at annotation boundaries. Together with the previously noted AT bias and RSCU skew, these findings indicate that the architectural framework of the mitochondrial genome is broadly stable, whereas evolutionary changes at the sequence and codon-usage levels are more likely to drive differentiation among populations or geographic lineages. Accordingly, structural rearrangements are unlikely to be the primary mechanism underlying mitochondrial diversification in this species, and subsequent comparative and selection analyses can focus—without confounding from structural change—on nucleotide substitutions, codon-usage bias, and the ratio of nonsynonymous to synonymous substitutions (dN/dS). A comparative analysis of the lengths of the 13 mitochondrial protein-coding genes (PCGs) across 18 mitogenomes revealed highly consistent gene sizes among samples, with only minor variation detected in a few loci (notably ATP8, ND3, and ND6). ND5 was the longest gene, whereas ATP8 was the shortest, and the lengths of the COX subunits and CYTB were particularly invariant. These patterns indicate that the coding regions are under strong functional constraint and are overall conserved; the small length differences observed are likely attributable to short indels within AT-rich segments and to differences in start/stop codon usage or annotation boundaries. Consistent with our observations of AT bias and codon-usage skew, these results suggest that mitochondrial divergence in this taxon is driven more by subtle sequence and codon-level evolution than by changes in genome architecture (Figure 4).

**TABLE 3:** Composition and skew in the mitochondrial genome of honey bee *A. cerana*-Southern China.

*A. cerana*-southern China	Size (bp)	A%	T%	G%	C%	A + T%	AT skew	GC skew
mitochondrial genome	16137	42.41	41.82	6.18	9.59	84.23	0.01	−0.22
*ND2*	996	39.46	47.19	5.12	8.23	86.65	−0.09	0.23
*COX1*	1566	34.67	41.44	11.05	12.84	76.11	−0.09	0.07
*COX2*	681	38.33	40.23	8.81	12.63	78.56	−0.02	0.18
*ATP8*	162	48.15	39.51	3.70	8.64	87.66	0.10	0.40
*ATP6*	678	36.58	47.35	5.60	10.47	83.93	−0.13	0.30
*COX3*	780	36.03	44.35	8.85	10.77	80.38	−0.10	0.10
*ND3*	354	37.29	48.02	5.09	9.60	85.31	−0.13	0.31
*ND5*	1668	46.82	37.89	5.70	9.59	84.71	0.11	0.25
*ND4*	1329	49.21	36.04	5.42	9.33	85.25	0.15	0.27
*ND4L*	264	52.65	34.47	3.41	9.47	87.12	0.21	0.47
*ND6*	513	42.88	43.08	5.46	8.58	85.96	0.00	0.22
*CYTB*	1149	36.64	44.39	8.53	10.44	81.03	−0.10	0.10
*ND1*	915	47.98	35.52	5.57	10.93	83.5	0.15	0.32
PCGs	11055	41.52	41.18	6.95	10.35	82.70	0.00	−0.20
tRNA genes	1486	45.09	42.26	5.52	7.13	87.35	0.03	−0.13
rRNA genes	2097	40.63	41.73	5.96	11.68	82.36	−0.01	−0.32
AT-rich region	818	47.43	50.00	0.98	1.59	97.43	−0.03	−0.24

### 3.3 rRNA and tRNA genes

**FIGURE 5:**
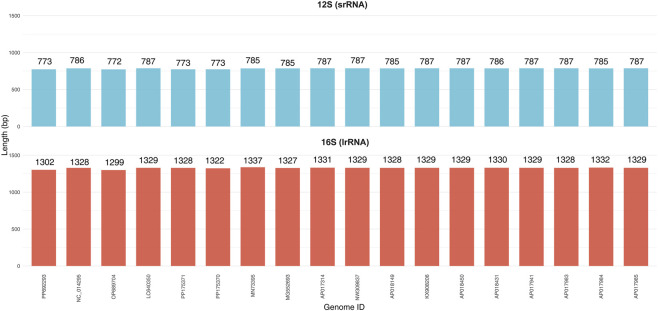
Length comparison of 12S (srRNA) and 16S (lrRNA) across 18 mitogenomes.

The *lrRNA* and *srRNA* of *A. cerana*-Southern China were 1324 bp and 773 bp in length, respectively. *lrRNA* and *srRNA* were typically separated by trnV (Table 2). The A + T and G + C content of rRNA genes were 82.36% and 17.64%, and the AT skew and GC skew were −0.01 and −0.32, respectively, suggesting an apparent bias toward the use of T and C. Based on the length comparison (Figure 5), the srRNA is predominantly 772–787 bp and the lrRNA 1299–1337 bp, with minimal variation across the 18 mitogenomes, further supporting strong structural conservation of the rRNA region; the two rRNAs are typically separated by *trnV*.

**FIGURE 6:**
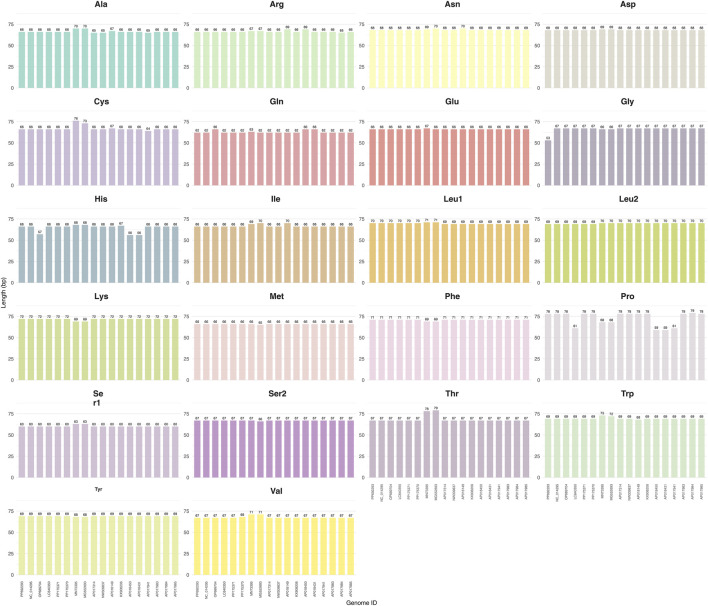
Length distribution of the 22 mitochondrial tRNA genes across 18 mitogenomes.

**FIGURE 7:**
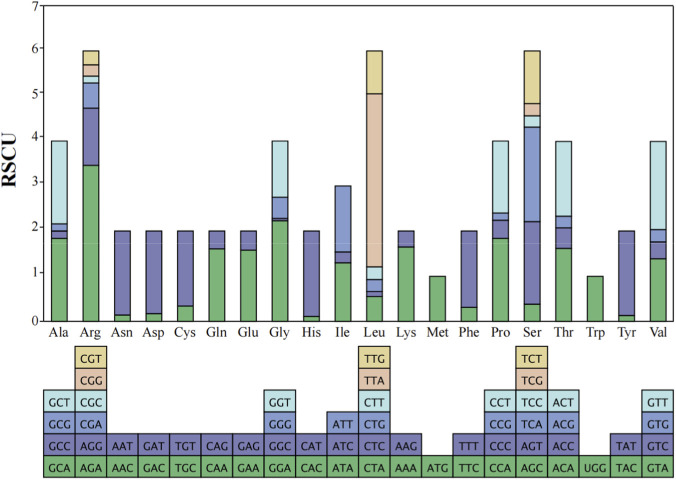
Codon bias statistics for 13 protein-coding genes of *A. cerana*-Southern China Ribosomal RNA genes and transfer RNA genes.

**FIGURE 8:**
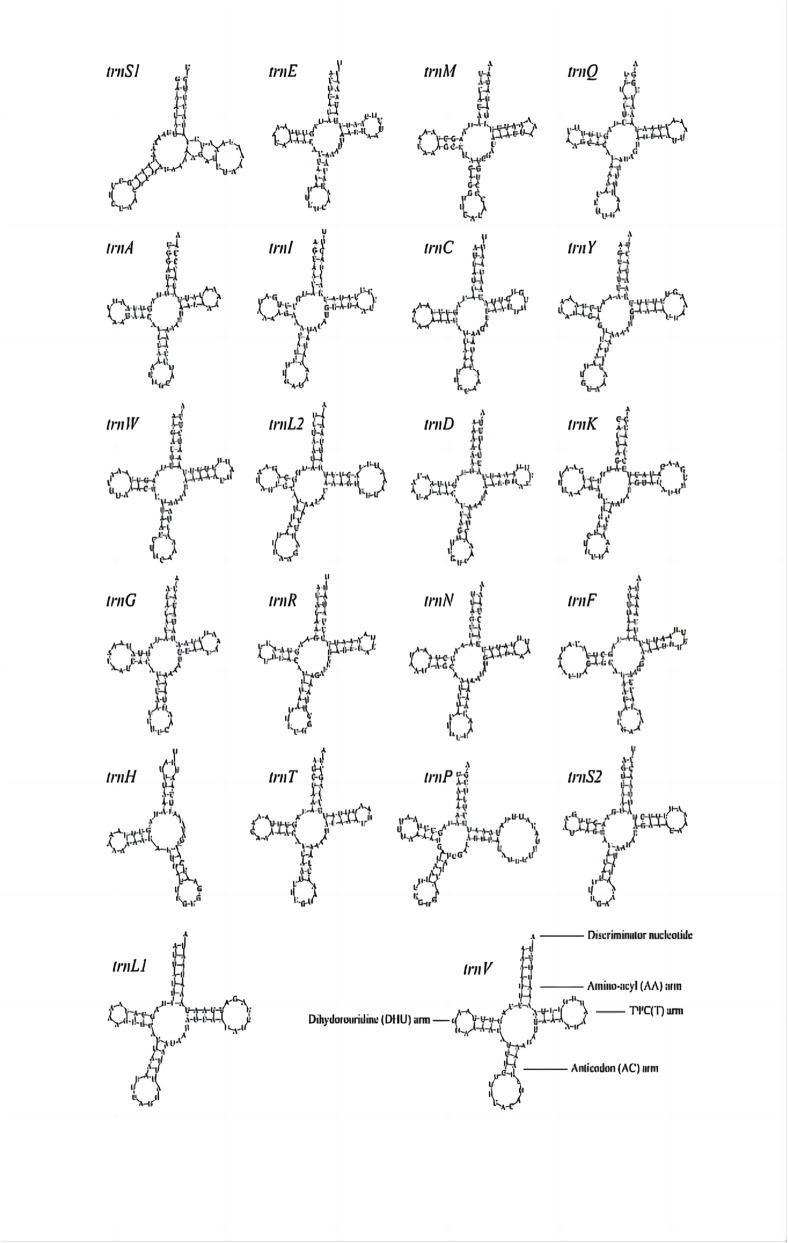
Predicted secondary structures for the tRNA genes in the *A. cerana*-Southern China mitochondrial genome.

In the mitochondrial genome of *A. cerana* (Southern China population), twenty-two tRNA genes were identified. Fourteen are encoded on the J-strand (major strand) and eight on the N-strand (minor strand). Except for *trnS1*, which lacks the dihydrouridine (DHU) arm, all tRNAs exhibit canonical cloverleaf secondary structures (Figures 6,7). The tRNA complement spans 1,468 bp in total, accounting for 9.10% of the mitogenome, with individual gene lengths ranging from 62 bp (*trnS1*) to 79 bp (*trnP*) (Table 3). Across genomes, tRNA lengths are tightly clustered—mostly 65–72 bp—with only minor 1-3 bp differences at a few loci, most notably in *trn-Pro* and *trn-Thr*; this pattern is consistent with small indels in AT-rich segments, subtle annotation-boundary shifts, and the missing DHU arm in *trnS1* (Figure 8). These regions are strongly AT-rich (A + T = 87.35%, G + C = 12.65%; A + T is 6.91-fold higher than G + C). The AT-skew and GC-skew are 0.03 and −0.13, respectively, indicating a slight bias toward A over T and a pronounced bias toward C over G.

### 3.4 AT-rich region

The AT-rich region of the *A. cerana*-Southern China mitochondrial genome extended over 818 bp and was located between *srRNA* and trnS1. The AT-rich region contained the highest A + T content (97.43%) in the entire mitogenome. Both the AT skew and GC skew for the AT-rich region were negative, indicating that T and C are more abundant than A and G, respectively (Table 3).

### 3.5 Phylogenetic analysis

**FIGURE 9:**
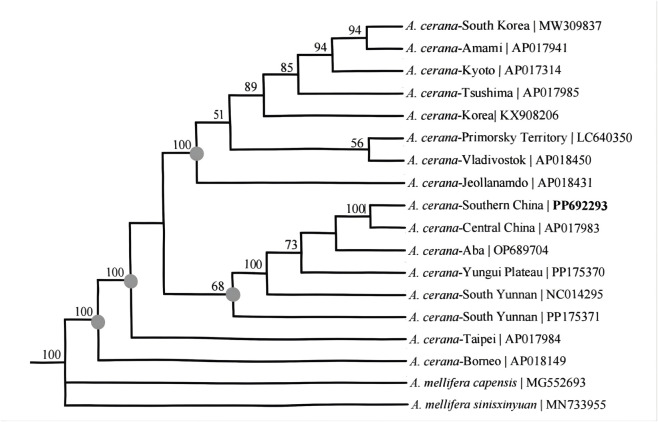
Phylogenetic tree of honey bee, *A. cerana* populations. *A. mellifera sinisxinyuan* and *A. mellifera capensis* as outgroups. The numbers on the branch points of the phylogenetic tree represent bootstrap values for the tree or evolutionary branching length.

Phylogenetic analysis was conducted using 13 PCGs with 17 closely related taxa (Figure 9). The results of the evolutionary tree analysis revealed that *A. cerana* was divided into four branches, one from Taiwan and one from Borneo, with high node support. *A. cerana* from Korea, Japan, and Russia formed a single colony, while those from mainland China formed an additional colony. *A. cerana*-Southern China and other *A. cerana* in mainland China represent sister groups. The genetic distance of *A. cerana*-Southern China was closer to that of *A. cerana*-Central China and *A. cerana*-Aba, and the differentiation time for *A. cerana*-South Yunnan was earlier.

### 3.6 Genome-wide Distribution of Nuclear Mitochondrial DNA segments (NUMTs)

**FIGURE 10:**
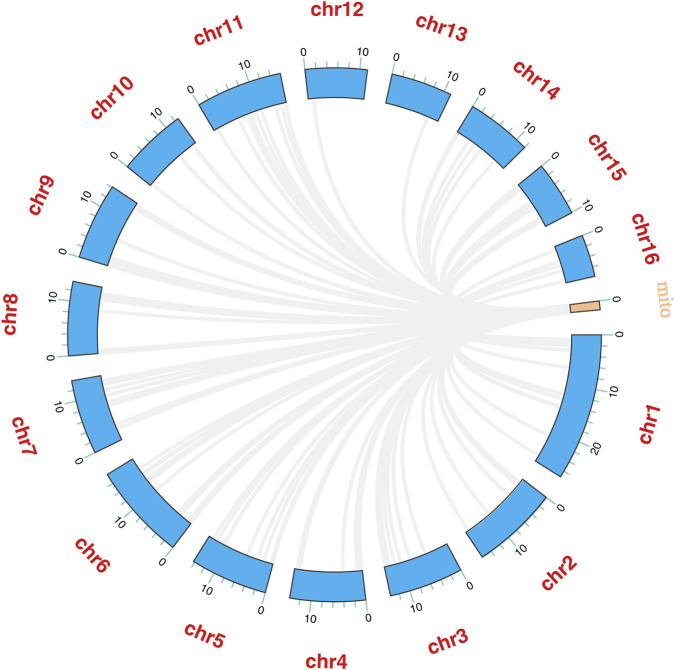
Genome-wide distribution of nuclear mitochondrial DNA segments (NUMTs) shown by Circos.

The circular Circos visualization shows mitochondrial homologous fragments mapping, via multiple links, to numerous loci on chr1–chr16, forming a multi-chromosomal, dispersed distribution rather than a continuous large syntenic block. The hits are predominantly short tracts (thin links with no extensive overlap), with relatively denser connections on chr1, chr6, and chr15–16. No long, one-to-one syntenic structure of mitochondrial coding regions was detected in the nuclear genome. This distribution pattern is consistent with nuclear mitochondrial DNA segments (NUMTs) (Figure 10).

## 4 Discussion

As populations of *A. cerana* continue to decline, it is vital to search for effective genetic markers to target and protect different types of *A. cerana*. Mitochondrial DNA is inherited maternally in bees, so a single bee can be used to represent an entire colony. Here, we report the complete mitochondrial genome of *A. cerana*-Southern China in Guangdong Province, China, which improve the data for mitochondrial molecular markers and comparative genomics of honey bee species.

Advances in sequencing technologies have enabled researchers to explore the complex genetic structure of *A. cerana* more accurately (Li et al., 2019; Okuyama et al., 2017). Compared with first-generation sequencing and next-generation sequencing, third-generation sequencing provides longer read lengths, which offer clear advantages for specific applications (Fan et al., 2020). In this study, we utilized PacBio HiFi sequencing to assemble the complete mitochondrial genomes of *A. cerana*-Southern China. Sequencing depth across the mitogenome was sufficient and broadly uniform, with an average coverage of 57.69× (range 30×–80×), supporting a high-confidence assembly (Supplementary Figure S1). Local fluctuations near AT-rich segments are expected for insect mitogenomes and did not affect gene recovery. According to our results, the full length of the *A. cerana*-Southern China mitochondrial genome was 16,137 bp, which is consistent with the previously reported total length of animal mitochondrial genomes ranging from 13 to 20 kb (Cameron, 2014). The mitochondrial genomes contain 37 common genes, including 13 PCGs, 22 tRNA genes, and 2 rRNA genes. All 37 mitochondrial genes exhibit a reversal of strand asymmetry on the majority strand, possibly due to the inversion of the replication origin located in the control region (Wei et al., 2010). tRNA genes and PCGs rearrangement have been previously reported in Hymenoptera (Su et al., 2023). However, this study of *A. cerana*-Southern China showed no rearrangement, which is considered to indicate the ground pattern of insect mitochondrial genomes. In most metazoan mitochondria, trnS1 generally has an unpaired DHU arm, while trnS2 has a standard cloverleaf structure. The *A. cerana*-Southern China trnS1 and trnS2 structures conform to this interpretation (Tan et al., 2011). Whether sequences tend to be A-encoded or T-encoded depends on the role they play. Strand asymmetry, also known as strand compositional bias, is typically indicated by the AT and GC skews (Perna and Kocher, 1995). We performed a codon preference analysis on 13 PCGs. The preference for the utilization of these synonymous codons is determined by several factors, including the abundance of tRNA, the mutational bias of the gene chain, gene expression level, gene length, and GC composition (Li et al., 2023). Our results show that the mitochondrial genome of *A. cerana*-Southern China favors NNA-type codons, which is consistent with the results of previous studies on *Apis* (Tang et al., 2023). Pairwise dN/dS (Ka/Ks) estimates for the 13 PCGs were all <1, indicating pervasive purifying selection (Supplementary Figure S2). Relatively elevated ratios were observed for *ND1*, *ND3*, and *CYTB* compared with *COX* genes, suggesting heterogeneous selective constraints among complexes.

Mitochondrial molecular markers provide a better understanding of the genetic structure and evolutionary history of honey bee populations, so they are widely used to study the biological origin, evolution, and genetic diversity of honey bees (Feng et al., 2024). The combination of sequencing technology and bioinformatics methods has advanced technological progress in the study of bee genome variation and environmental adaptive genome characteristics. At present, the genes commonly used for sequencing and analysis in honey bee mitochondrial genome research include tRNA^leu^-*COII*, *COII*, *lrRNA*, *CYTB* and *ND2*. In addition, we found that the length of the AT-rich region of *A. cerana* is very different among different groups, and the AT-rich region may be a better subspecies division as a mitochondrial molecular marker fragment (Zhang et al., 2023). This region can also reflect the adaptive evolution of *A. cerana* to their living environment, and researchers are investigating the impact of this complex region on the genetic differentiation of species (Liu et al., 2014).

Phylogenetic analysis was conducted using the 13 PCGs with 17 closely related taxa. The results further suggest that the current subspecies structure is the result of multiple population divisions that occurred in a common ancestor. The results showed that all genome sequences of *A. cerana* were mainly divided into four branches. *A. cerana* from Taiwan and Borneo form their own branch. The sequences from Russia, Japan and Korea were clustered in a single group, which was consistent with a previous report (Xu et al., 2024). The genetic distance between *A. cerana*-Southern China, *A. cerana*-Central China and *A. cerana*-Aba close. This was followed by *A. cerana*-South Yunnan and *A. cerana*-Yungui Plateau. However, compared to these types of *A. cerana*, the differentiation time of *A. cerana*-South Yunnan was the earliest. However, there appeared to be no correlation between genetic diversity and geographical distance. Morphometric analysis revealed that *A. cerana* exhibits high diversity in mountainous regions and islands, a consequence of selection and genetic drift due to prolonged isolation (Ji et al., 2023; Yu et al., 2019). Previous study on honeybees in various regions, including an analysis of morphological differences between *A. cerana*-South Yunnan and *A. cerana*-Yungui Plateau, have indicated that the Tropic of Cancer serves as a geographic boundary (Zhang et al., 2014). The analysis of the correlation between the morphological indexes of *A. cerana* and the climate factors of their living environment found that most of the classical morphological characteristics of *A. cerana*, such as body size and wing length, were significantly correlated with climate characteristics (Qiu et al., 2023). This suggests that there are differences in the genetic distance of *A. cerana*, even when the physical distance is close.

The populations of *A. cerana* are prone to genetic differentiation and exhibit a high level of diversity. In contrast to previous studies that provided only limited information about *A. cerana* at the genetic level, the mitochondrial genome-wide analysis of *A. cerana* in our study offers a more detailed genetic structure of honeybees. The effective population size and genetic diversity of *A. cerana* in China are high, making the populations and the species within this range in general vulnerable to the effects of genetic drift and selection. Due to human and environmental damage, this could lead to the extinction of local populations. Therefore, further research is required to determine the optimal approach for conserving the genetic resources of specific *A. cerana* populations. In this paper, the study of the mitochondrial genome of *A. cerana* may be limited. Its genetic information needs further exploration, possibly in conjunction with other data, such as bee morphology in different environments. This approach would lead to a more comprehensive understanding of its biological characteristics and evolutionary history.

## 5 Conclusion

In conclusion, we assembled a high-quality mitochondrial genome of *A. cerana*-Southern China using PacBio HiFi sequencing technology. We have detailed the mitochondrial genome information of *A. cerana*-Southern China. Phylogenetic studies have indicated that the mitochondrial genome is a reliable molecular marker for studying the phylogeny of *A. cerana*. By comparing the mitochondrial genomes, we found that *A. cerana* exhibits high genetic diversity at the mitochondrial genomic level across different environments. The results of the phylogenetic tree indicated that the differentiation time of *A. cerana*-Southern China was the latest compared to other sister groups. This study provided data for the gene bank of *A. cerana*. It is hoped that more specimens can be collected to conduct a more comprehensive and systematic study of honeybees. The morphological data and gene fragments can be utilized for combined analyses in the future.

## Data Availability

The datasets presented in this study can be found in online repositories. The names of the repository/repositories and accession number(s) can be found below: https://www.ncbi.nlm.nih.gov/genbank/, PP692293.
