# Supplementary figures and images for: The complete mitochondrial genome of Apis cerana-southern China (Hymenoptera: Apidae) and insights into the phylogenetics
Source: Front Genet. 2026 Jan 21;16:1737945. doi: 10.3389/fgene.2025.1737945 (PMC12867336; doi:10.3389/fgene.2025.1737945)

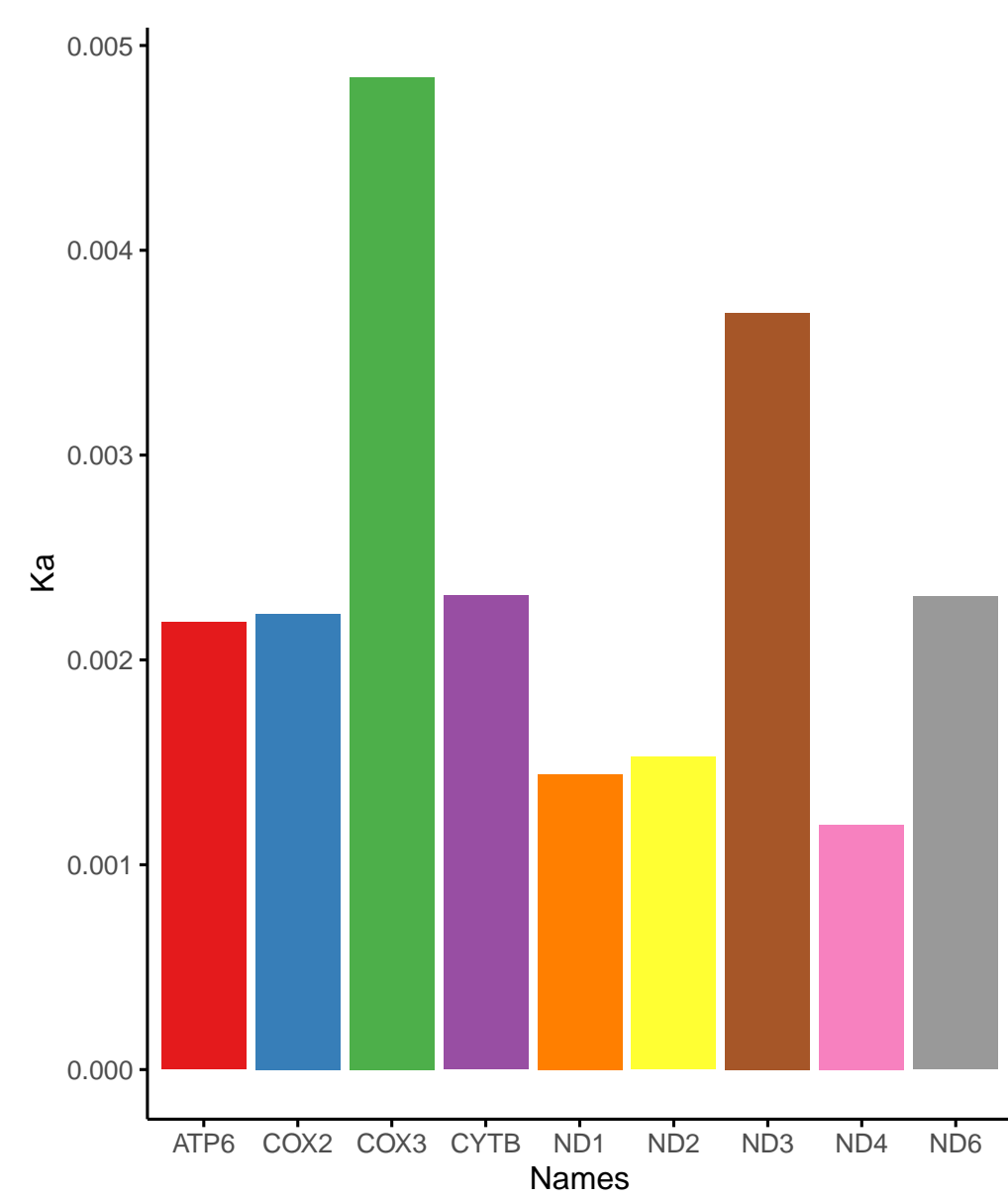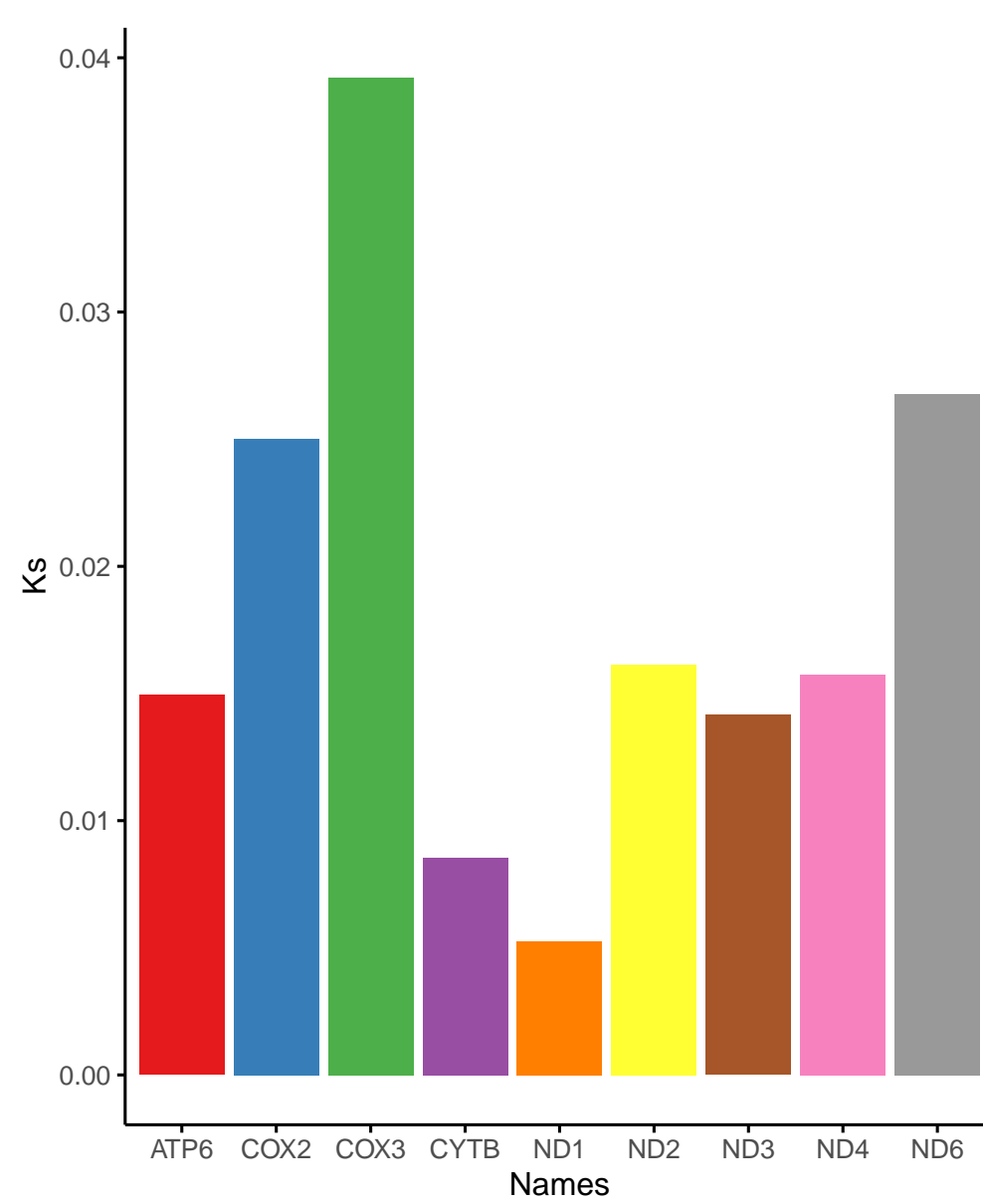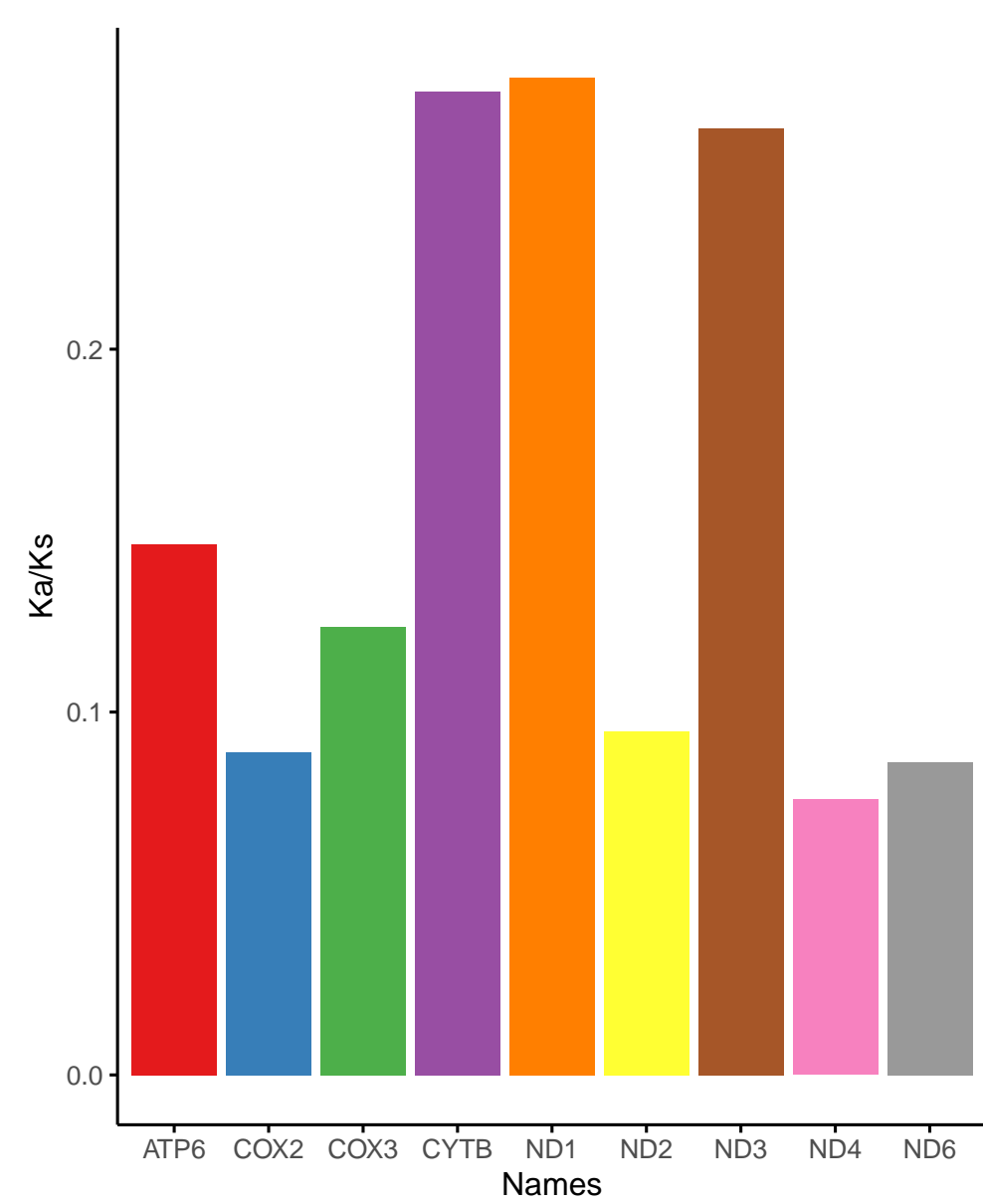

Supplement: Supplementary file 1 [file DataSheet2.pdf]
