# Supplementary material for: The complete mitochondrial genome of Apis cerana-southern China (Hymenoptera: Apidae) and insights into the phylogenetics
Source: Front Genet. 2026 Jan 21;16:1737945. doi: 10.3389/fgene.2025.1737945 (PMC12867336; doi:10.3389/fgene.2025.1737945)
Supplement: Supplementary file 2 [file DataSheet1.pdf]

Sequencing Depth and Coverage Map

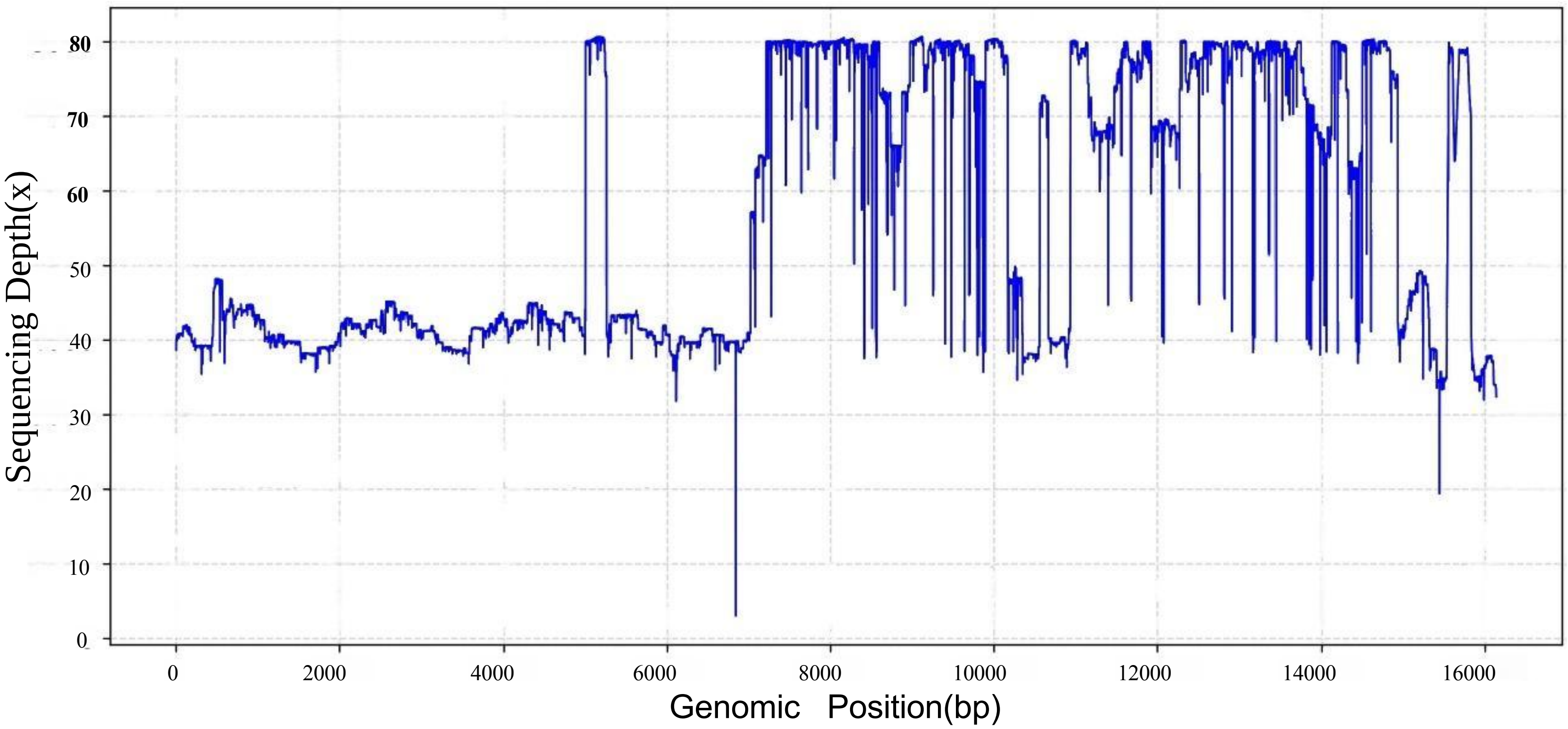

(1)Total genome length=16,137 bp (2)Average depth=57.69×

(3)Maximal depth=80× (4)Minimal depth=30×
